# Comparison of *In Vitro* Fertilization/Intracytoplasmic Sperm Injection Cycle Outcome in Patients with and without Polycystic Ovary Syndrome: A Modified Poisson Regression Model 

**DOI:** 10.22074/ijfs.2018.5117

**Published:** 2017-10-14

**Authors:** Amir Almasi-Hashiani, Mohammad Ali Mansournia, Mahdi Sepidarkish, Samira Vesali, Azadeh Ghaheri, Arezoo Esmailzadeh, Reza Omani-Samani

**Affiliations:** 1 Department of Epidemiology and Reproductive Health, Reproductive Epidemiology Research Center, Royan Institute for Reproductive Biomedicine, ACECR, Tehran, Iran; 2 Department of Epidemiology and Biostatistics, School of Public Health, Tehran University of Medical Sciences, Tehran, Iran; 3 Department of Obstetrics and Gynecology, Zahedan University of Medical Sciences, Zahedan, Iran

**Keywords:** Intracytoplasmic Sperm Injection, *In Vitro* Fertilization, Polycystic Ovary Syndrome, Pregnancy

## Abstract

**Background:**

Polycystic ovary syndrome (PCOS) is a frequent condition in reproductive age women with a prevalence rate of 5-10%. This study intends to determine the relationship between PCOS and the outcome of assisted reproductive treatment (ART) in Tehran, Iran.

**Materials and Methods:**

In this historical cohort study, we included 996 infertile women who referred to Royan Institute (Tehran, Iran) between January 2012 and December 2013. PCOS, as the main variable, and other potential confounder variables were gathered. Modified Poisson Regression was used for data analysis. Stata software, version 13 was used for all statistical analyses.

**Results:**

Unadjusted analysis showed a significantly lower risk for failure in PCOS cases compared to cases without PCOS [risk ratio (RR): 0.79, 95% confidence intervals (CI): 0.66-0.95, P=0.014]. After adjusting for the confounder variables, there was no difference between risk of non-pregnancy in women with and without PCOS (RR: 0.87, 95% CI: 0.72-1.05, P=0.15). Significant predictors of the ART outcome included the treatment protocol type, numbers of embryos transferred (grades A and AB), numbers of injected ampules, and age.

**Conclusion:**

The results obtained from this model showed no difference between patients with and without PCOS ac- cording to the risk for non-pregnancy. Therefore, other factors might affect conception in PCOS patients.

## Introduction

The International Committee for Monitoring Assisted Reproductive Technology and the World Health Organization (WHO) define infertility as the failure to achieve a clinical pregnancy for 12 months or more of regular unprotected intercourse (1-3). Infertility is recognized as one of the main public health concerns by WHO (4, 5). There are 72.4 million (low estimate: 40.2 million, high estimate: 120.6 million) currently infertile women aged 20-44 years, from which 10.98 million reside in more developed countries and 61.4 million reside in less developed countries (5). The National Infertility Study (2004- 2005) has reported that Iran, as a less developed country, has a prevalence rate of current primary infertility of 3.4% and lifetime primary infertility of 24.9% (6).

The prevalence of infertility is increasing worldwide; hence, assisted reproductive technology (ART) and its success rate are critical (7). Infertility is a multi-factorial disorder with different etiologies. One cause is polycystic ovary syndrome (PCOS) (8). PCOS is one of the most frequent hormonal disorders among reproductive age women with a prevalence rate of 5-10%. PCOS consists of reproductive, metabolic, and cardiovascular components (9). The prevalence rate of PCOS in Iran has been determined as 7.1% by the National Institute of Health (NIH) definition, 11.7% by the Androgen Excess Society criteria (AES), and 14.6% according to the Rotterdam consensus (Rott) (10). PCOS is one of the prevalent causes of infertility due to anovulation (11). Approximately 90 to 95% of women who refer to infertility clinics with anovulation have PCOS (12-14). Because the etiology of PCOS is not defined, its therapy remains mostly symptomatic and empirical (15). An increased risk gestational diabetes and other pregnancy complications has been shown in women with PCOS (16). Previous studies reported no difference between women with and without PCOS in clinical pregnancy rate (PR) (17-19). This study aimed to determine the association between PCOS and *in vitro* fertilization/ intracytoplasmic sperm injection (IVF/ICSI) cycle outcome in infertile women who referred to Royan Institute, Tehran, Iran between January, 2012-December, 2013.

## Materials and Methods

This historical cohort study consisted of infertile women who referred to Royan Institute for Reproductive Biomedicine, Stem Cell Biology and Technology Center, Tehran, Iran between January 2012 and December 2013. Patients were recruited according to their medical records in the institute’s files. The study population consisted of subjects who presented with primary or secondary infertility that visited the clinics more than once and received IVF/ICSI treatment regardless of the cause of infertility.

We defined the relationship between PCOS status and clinical pregnancy based on clinical criteria. This cohort study was divided into 2 groups: women with and without PCOS. The outcome variable was clinical pregnancy, which was determined as the presence of a fetal heartbeat by ultrasound performed at 6 to 8 weeks after the last menstrual period. Diagnosis of PCOS was established based on the presence of two of the following Rott criteria: oligomenorrhea (defined by more than six cycles with a length of more than 35 days) and/or anovulation; clinical (presence of hirsutism with a score of 8 or more, persistence of acne during the third decade of life or later, or the presence of androgenic alopecia); biochemical signs of hyperandrogenism; and polycystic ovaries (PCO) visualized on ultrasound with the presence of 12 or more follicles that measured 2-9 mm in diameter in each ovary and/or ovarian volume more than 10 cm^3^.

The potential confounding factors that we considered in the analysis were: treatment modality (IVF/ICSI); unusual vaginal discharge (diagnosed based on clinical symptoms and sexual history); hyperandrogenism; ART protocol (long, antagonist, short, other); history of ART cycle; age; body mass index (BMI); number of grade A embryos transferred; number of grade AB embryos transferred based on an evaluation of the number of blastomeres, blastomere symmetry, and the number of anucleated fragments; the number of injected ampules; human chorionic gonadotropin (hCG) administration day; and number of oocytes injected. These variables were recorded in the patients’ medical files.

### Statistical analysis

The study was designed to have 80% power to detect a 15% difference in clinical pregnancy, with two-sided alpha levels of 0.05. According to sample size calculation for independent proportions there should be a minimum of 150 participants in each group. Descriptive data was expressed as percentages, mean, and SD. First, we examined the individual association of the PCOS status and pregnancy failure by log binomial regression model; next, any potential confounder predictors were entered into the model. Convergence problems may arise with binomial regression models, therefore we modeled the outcome by Poisson Regression Model. On the other hand, use of Poisson regression tends to provide conservative results. Finally, we have used a modified Poisson regression model to estimate the risk ratios (RRs) and 95% confidence intervals (CIs) after adjustment for potential confounders. Royston fractional polynomials (20, 21) were used for all continuous measures in the model in order to examine the proper scale of variables. Stata software (version 13, Stata Corp., College Station, TX, USA) was used for all statistical analyses.

The Board of Research Ethics at Royan Institute approved this study (code: EC/89/1046). All subjects provided informed consent to participate and they received assurance that the results would be published statistically without any personal data.

## Results

Out of 996 infertile women who referred to Royan Institute as a referral center for ART in Iran, 13 (1.31%) were lost to follow up and 983 cases were successfully followed. The cases appropriately followed had a PR of 35.20%. Patients underwent ultrasound imaging (sonography) at the start of the treatment process, which was administered by a radiologist. The results showed that 115 (11.72%) had PCOS. The remaining 866 (88.28%) cases did not have PCOS. The mean (SD) age of clinically pregnant women was 34.27 (4.92) years. The mean age for the non-pregnant group was 36.15 (5.43) years. The difference in the mean age was statistically significant (P=0.001). There was no meaningful difference (P=0.90) in BMI of clinically pregnant (25.54 kg/m^2^, SD: 3.92) compared to non-pregnant women (25.57 kg/m^2^, SD: 3.85 (Table 1).

There was no significant association between infectious discharge, stimulator drug type, hyperandrogenism, history of ART cycle, BMI, hCG administration day, the injected oocyte number, and ART cycle outcome. The results showed a negative association between age and the injected ampule number, and risk of non-pregnancy. Cases that underwent the long protocol had a better outcome. The numbers of grade A and AB embryos transferred, after adjustment for potential confounders, showed a negative association with ART cycle outcome. Fractional polynomial regression was checked for age, BMI, ampule number, hCG day, and injected oocyte number. The results indicated that the linear form was the best scale for these continuous variables. Crude analysis revealed that the risk of non-pregnancy in women who underwent ART in patients with PCOS was 0.53%; for those without PCOS, it was 0.66% (RR: 0.79, 95% CI: 0.66-0.95, P=0.014). Hence, there was a significantly lower risk of non-pregnancy in PCOS cases compared to cases without PCOS. After adjustment for confounder variables (Table 2), we observed no difference between the risk of nonpregnancy in women with and without PCOS (RR: 0.87, 95% CI: 0.72-1.05, P=0.15).

**Table 1:** A comparison of independent variables based on outcome status


Variable		Clinical pregnancy	Non-pregnancy	P value

PCOS^¶^				0.006
	No	291 (33.60)	575 (66.40)	
	Yes	54 (46.96)	61 (53.04)	
Stimulate medicine ^¶^				
HMG		29 (25.66)	84 (74.34)	0.001
Pure FSH		34 (37.78)	56 (62.22)	
Gonal F		114 (48.31)	122 (51.69)	
HMG+Gonal F		121 (28.34)	306 (71.66)	
Others		47 (41.23)	67 (58.77)	
Infectious discharge ^¶^				
Yes		59 (32.78)	121 (67.22)	0.462
No		286 (35.66)	516 (64.34)	
ART protocol ^¶^				
Long		290 (37.37)	486 (62.63)	0.001
Antagonist		35 (28.46)	88 (71.54)	
Short		4 (10.81)	33 (89.19)	
Other		5 (18.52)	22 (81.48)	
Hyperandrogenism ^¶^				
Hirsutism		38 (43.18)	50 (56.82)	0.102
None		307 (34.34)	587 (65.66)	
History of ART cycle^¶^				0.217
	Yes	210 (33.76)	412 (66.24)	
	No	136 (37.67)	225 (62.33)	
Age (Y)^*^		34.27 (4.92)	36.15 (5.43)	0.001
BMI^*^		25.54 (3.92)	25.57 (3.85)	0.908
Embryo transferred grade A^*^		0.26 (0.68)	0.13 (0.46)	0.001
Embryo transferred grade AB^*^		0.97 (1.07)	0.63 (0.92)	0.001
Ampule number^*^		26.23 (10.01)	30.82 (14.18)	0.001
hCG administration day^*^		12.38 (2.64)	12.35 (2.49)	0.878
Injected oocyte number^*^		8.12 (3.90)	7.03 (4.24)	0.001


^¶^; Number (%), *; Mean (SD), PCOS; Polycystic ovary syndrome, ART; Assisted reproductive treatment, BMI; Body mass index, hCG; Human chorionic gonadotropin, HMG; Human menopausal gonadotropin, and FSH; Follicle-stimulating hormone.

**Table 2:** Crude and adjusted RR for factors associated with clinical pregnancy (dependent variable) which remained in the final modified Poisson regression model


Variable	Crude estimate			Adjusted estimate^*^			
	RR	95% CI	P value		RR	95% CI	P value

PCOS	0.79	0.66-0.95	0.014		0.87	0.72- 1.05	0.15
Age (Y)	1.02	1.01-1.03	0.001		1.01	1.01-1.02	0.001
Embryo transferred grade A	0.84	0.75-0.94	0.004		0.80	0.71-0.90	0.001
Embryo transferred grade AB	0.87	0.83-0.92	0.001		0.86	0.82-0.91	0.001
Infectious discharges	0.95	0.85-1.07	0.45		0.91	0.81-1.01	0.10
ART protocol	1.12	1.07-1.18	0.001		1.06	1.01-1.11	0.02
Stimulator drug type	0.99	0.95-1.03	0.82		0.97	0.93-1.01	0.16
Ampule number	1.008	1.006-1.011	0.001		1.004	1.001-1.007	0.008


*; Adjusted for the other variables in the Table, PCOS; Polycystic ovary syndrome, ART; Assisted reproductive treatment, RR; Risk ratio, and CI; Confidence interval.

## Discussion

The present study measured pregnancy outcome in PCOS and non-PCOS infertile patients who underwent IVF/ICSI. A number of studies investigated the pregnancy outcome in PCOS women who underwent infertility treatments. Most studies were performed in Western countries. Our study differed from other reports in several key ways. First, we have performed this study in one of the countries in Asia, located in the Middle East. Asian women are slightly distinct from Western women in terms of PCOS, obesity, and their complications (22). In the current study, the clinical pregnancy was considered the ART outcome in a comparison between PCOS and non-PCOS infertile women treated by IVF/ICSI. A large number of studies attempted to determine other indicators (implantation and live birth rates) in addition to the clinical PR in infertile women with and without PCOS (23). The effectiveness of a novel modified ART protocol compared with a conventional ART protocol, ultra-long agonist (ULA) protocol versus long agonist (LA) protocol, and gonadotropin-releasing hormone (GnRH) antagonist versus GnRH agonist long protocols on PCOs females who underwent ART has been evaluated in a few studies (17, 24, 25).

Retrospective and prospective researches have reported adverse pregnancy outcomes that include miscarriage rate, multiple PR, abortion rate (26), prevalence of preterm delivery, and pregnancy induced hypertension in these women (22). However, the distinction or novelty of the current study was the use of modified Poisson regression when the outcome of interest (clinical pregnancy) was dichotomous. In an assessment of risk of pregnancy failure, interpretation of the odds ratio, obtained from logistic regression, as a RR leads to its potential exaggeration. Numerous reports have proposed that the RR is preferred over the odds ratio for most prospective investigations. It was demonstrated that application of logistic regression to prospective studies is uncritical and naive conversion of an adjusted odds ratio to a RR has compounded the difficulties, such as invalid confidence limits and inconsistent estimates for the RR, which was not reduced with increasing sample size (15, 27).

Our findings showed no significant difference in PR in patients with and without PCOS who underwent IVF/ ICSI. We obtained this result after adjustments for confounder variables of age, type of ART protocol, and quality of the transferred embryo. A study on 189 infertile patients with PCOS, 129 PCOS, and 142 without PCOS or PCOS (control) who underwent IVF-ET indicated no significant differences in the clinical PR between the PCOs group (202 cycles, 51.0%), PCO group (134 cycles, 53.0%), and control group (150 cycles, 46.0%) (18). Another study analyzed clinical and biological features of PCOS in patients enrolled in ICSI cycles compared to normo-ovulatory women. The clinical PR per transfer (31.5 vs. 22.2%, not significant) did not differ statistically in the two groups (19).

Findings obtained from a systematic review and metaanalysis of 7 published studies (755 patients) reported no significant difference in PR (OR=1.08, 95% CI: 0.80- 1.45) between the GnRH antagonist group and the GnRH agonist group (17). However, another study (24) evaluated the effectiveness of ULA and LA. The results indicated that the ULA protocol yielded significant higher clinic PR (70.2%) compared to the LA protocol (50.8%) in women with elevated BMI and PCOS who underwent IVF/ICSI. In the current study, the final modified Poisson regression model (adjusted model) demonstrated a significant difference in pregnancy outcome in patients who underwent IVF/ICSI according to the type of ART protocol used.

We were unable to follow up pregnancy complications, live births, and infants as other outcomes of ART. A longterm follow up of newborns of infertile patients who underwent IVF/ICSI, particularly PCOS women, should be performed in the future.

## Conclusion

This paper proposed the use of modified Poisson regression to estimate the risk of pregnancy failure. The results obtained from this model showed no difference between patients with and without PCOS according to the risk of non-pregnancy.
